# Eckol Enhances Heme Oxygenase-1 Expression through Activation of Nrf2/JNK Pathway in HepG2 Cells

**DOI:** 10.3390/molecules191015638

**Published:** 2014-09-29

**Authors:** Young-Jin Jun, Minsup Lee, Taisun Shin, Nayoung Yoon, Ji-Hoe Kim, Hyeung-Rak Kim

**Affiliations:** 1 Department of Food Science and Nutrition, Pukyong National University, Busan 608-737, Korea; E-Mails: jyj0127@pknu.ac.kr (Y.-J.J.); mlee15@lsuhsc.edu (M.L.); 2 Division of Food and Nutrition, Chonnam National University, Gwangju, 500-757, Korea; E-Mail: shints@jnu.ac.kr; 3 Food Safety Research Division, National Fisheries Research and Development Institute, 408-1 Sirang-ri, Gijang-eup, Busan 619-705, Korea; E-Mails: dbssud@korea.kr (N.Y.); kimjh@korea.kr (J.-H.K.); 4 Korea Ginseng Corporation Research Institute, Daejeon 305-805, Korea; E-Mail: jyj0127@kgc.co.kr

**Keywords:** eckol, *Ecklonia stolonifera*, heme oxygenase, Nrf2, JNK

## Abstract

Eckol isolated from *Ecklonia stolonifera* was previously reported to exhibit cytoprotective activity with its intrinsic antioxidant activity in *in vitro* studies. In this study, we characterized the mechanism underlying the eckol-mediated the expression of heme oxygenase-1 (HO-1). Eckol suppressed the production of intracellular reactive oxygen species and increased glutathione level in HepG2 cells. Eckol treatment enhanced the expression of HO-1 at the both level of protein and mRNA in HepG2 cells. Enhanced expression of HO-1 by eckol was presumed to be the activation of the nuclear factor erythroid-derived 2-like 2 (Nrf2) demonstrated by its nuclear translocation and increased transcriptional activity. c-Jun NH_2_-terminal kinases (JNKs) and PI3K/Akt contributed to Nrf2-mediated HO-1 expression. These results demonstrate that the eckol-mediated expression of HO-1 in HepG2 cells is regulated by Nrf2 activation via JNK and PI3K/Akt signaling pathways, suggesting that eckol may be used as a natural antioxidant and cytoprotective agent.

## 1. Introduction

Reactive oxygen species (ROS) are continuously produced in aerobic organisms as a natural by-product of oxygen metabolism and act as subcellular messengers in complex cellular processes, such as mitogenic signal transduction, gene expression, and regulation of cell proliferation. Excess ROS are involved in various pathological conditions, including aging, cancer, inflammation, and hepatic diseases, therefore, antioxidants to reduce ROS has been proposed to the prevention of diseases associated with oxidative damage [1,2,3,4]. For this, various phytochemicals possessing intrinsic antioxidant properties or triggering the intracellular cascade of protective signaling pathways may offer promising strategy for therapeutic applications for the diseases related to oxidative damage. A number of studies have been demonstrated the chemopreventive effects of phytochemicals, and have led to the discovery of new classes of chemopreventive agents, such as polyphenols, carotenoids, and organosulfur compounds. Exposure to the chemopreventive agents produces certain level of ROS, and causes mild oxidative stresses in cells [5]. Such mild oxidative stresses are sufficient to initiate the intracellular signaling lead to the induction of phase II detoxification enzymes and antioxidant enzymes [6,7]. As an antioxidant enzyme, heme oxygenase-1 (HO-1) catalyzes the rate-limiting step in toxic heme catabolism, leading to the formation of free iron, carbon monoxide (CO), and biliverdin, which in turn is reduced to bilirubin by biliverdin reductase. Many studies have demonstrated the potent antioxidant and cytoprotective activities of heme-derived metabolites produced by HO-1 catalysis [8,9].

A redox-sensitive transcription factor, nuclear factor erythroid-derived 2 (NF-E2)-like 2 (Nrf2), which is a member of the NF-E2 family of the basic leucine zipper, has been shown to play a critical role in deactivating ROS and carcinogens. Under physiological conditions, Nrf2 is inactive in the cytoplasm by sequestering Kelch-like ECH-associated protein 1 (Keap1), which inhibits the translocation of Nrf2 into the nucleus [10]. Thus, cytoplasmic Keap1 protein is a repressor of Nrf2-mediated antioxidant response element (ARE) activity. The activation of Nrf2-ARE pathway leads to induction of antioxidant enzyme, such as HO-1. Due to the potential role of Nrf2-ARE signaling pathway in inducing antioxidant enzymes and protecting cells against oxidative damage, there are growing interests in the molecules that could activate this pathway [11]. A large number of studies have demonstrated that phytochemicals, such as resveratrol [12], curcumin [13], epigallocatechin-3-gallate [14], and eckol [7], have cytoprotective effects by enhancing Nrf2-ARE pathway-mediated antioxidant enzymes in *in vitro* and *in vivo* experiments.

**Figure 1:**
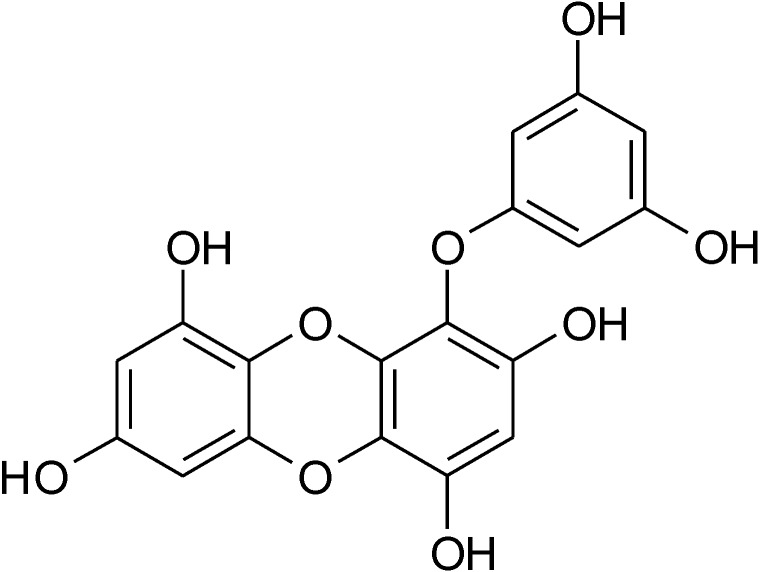
Chemical structure of eckol isolated from *Ecklonia stolonifera*.

*Ecklonia stolonifera* contains various kinds of biologically active phlorotannins, marine algal polyphenols in the form of phloroglucinol polymers. Among these phlorotannins, eckol (Figure 1) which is a trimer of phloroglucinol, has been reported to have antioxidant [15,16], acetylcholine esterase inhibitory [17], anti-inflammatory [18,19], and anti-diabetic activities [20]. As antioxidative mechanisms of eckol, the compound enhanced Nrf2-mediated HO-1 expression via activation of ERK and PI3K/Akt pathways in lung fibroblast [7]. However, little is known about cytoprotective roles of eckol through the regulation of Nrf2/HO-1 signaling pathways. In this study, we isolated eckol from *E. stolonifera* and evaluated its cytoprotective properties and the underlying mechanism of the activation of Nrf2 signaling pathway in human hepatoma cell line (HepG2).

## 2. Results and Discussion

### 2.1. Antioxidant Activity of Eckol

**Figure 2:**
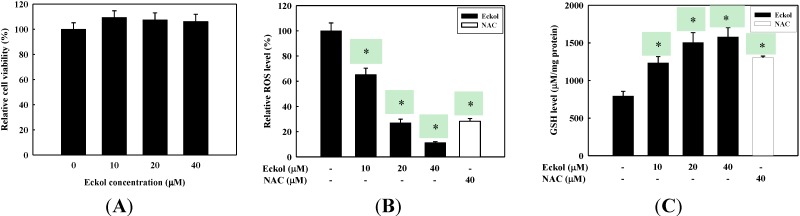
Effect of eckol on the inhibition of ROS production in HepG2 cells. (**A**) Cells were treated with eckol for 24 h at the indicated concentrations. The cell viability was determined by MTS assay kit; (**B**) The cells pretreated with indicated concentration of eckol or NAC (40 μM) for 2 h were stimulated with 0.5 mM H_2_O_2_ for 30 min. ROS levels were measured by DCF-DA with fluorescent analysis; (**C**) The cells were treated with indicated concentration of eckol or NAC (40 µM) for 2 h and cellular GSH was measured with assay kit. Values are the mean ± SD of three independent experiment. * *p* < 0.05 indicates significant differences compared to the control group.

The cytotoxicity of eckol was measured using MTS assays on HepG2 cells prior to testing of eckol for its suppression of ROS production. As shown in Figure 2A, eckol did not show cytotoxicity in HepG2 cells up to 40 μM concentration. ROS scavenging activity of eckol was assessed in order to confirm its antioxidant ability to suppress the production of free radicals in cellular environments. For this purpose, H_2_O_2_ was used as an oxidant for the generation of intracellular ROS. The antioxidant activity of eckol was compared with a known antioxidant, N-acetylcysteine (NAC), treated in the same assay. As shown in Figure 2B, eckol treatment inhibited the production of ROS in H_2_O_2_-treated HepG2 cells in a dose-dependent manner. The relative ROS level of 40 μM eckol was estimated to be 11.2% ± 0.85% compared to the non-treated group, which is much stronger ROS scavenging activity compared to NAC. Glutathione (GSH), a potent intracellular antioxidant, plays a primary role in the detoxification of various electrophilic compounds and peroxide, thus we assessed the effect of eckol on the intracellular GSH level. The intracellular GSH content in HepG2 cells was dose-dependently enhanced by eckol treatment (Figure 2C), indicating higher antioxidant activity compared with NAC.

The human hepatic HepG2 cell line has been widely used for studying *in vitro* oxidative damage and xenobiotic metabolism, since they maintain majority of specialized functions like normal human hepatocytes. In particular HepG2 cells maintain their endogenous expression of various antioxidative and xenobiotic metabolizing enzymes, and have been used as a model for studying the mechanisms of oxidative injuries and protection against oxidative stress [21]. The cytoprotective properties of antioxidants have been ascribed to their ability to increase the activities of intracellular antioxidant enzymes or to scavenge ROS directly as an electrophilic agent. As previous reports, antioxidant phlorotannins, such as 2-phloroeckol, eckol, and phlorofucofuroeckol A, showed hepatoprotective effect through direct scavenging of intracellular ROS in tacrine-treated HepG2 cells [22]. The antioxidant activity of eckol may be in part due to its direct scavenging intracellular ROS as well as enhancing intracellular GSH level.

### 2.2. Effect of Eckol on HO-1 Expression

**Figure 3:**
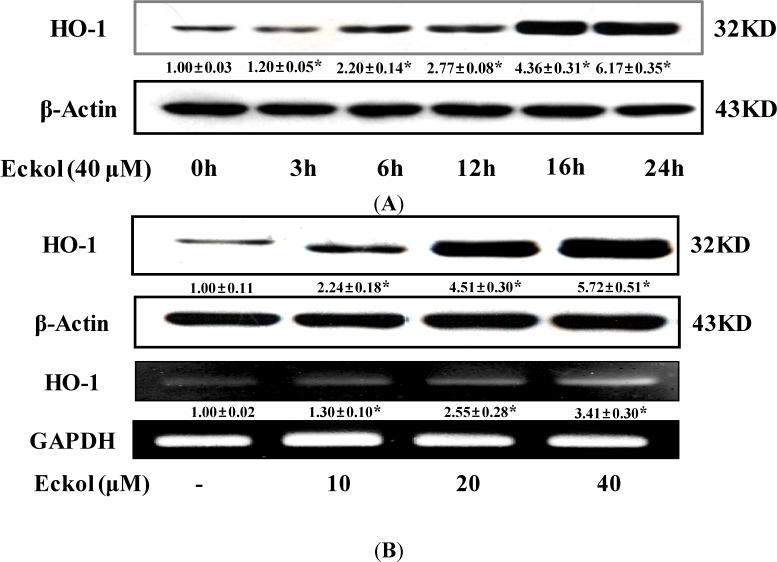
Effects of eckol on expressions of HO-1 in HepG2 cells. (**A**) Cells were treated with 40 μM eckol with indicated time and equal amounts of total proteins were subjected to SDS-PAGE; (**B**) Cells were treated with eckol for 16 hr and equal amounts of total proteins were subjected to SDS-PAGE. The expressions of HO-1 and β-actin protein were detected by Western blot using specific antibodies. Isolated RNA was reverse-transcripted and amplified by PCR using specific primers. Values are the mean ± SD of three independent experiments. * *p* < 0.05 indicates significant differences compared to the control group.

To determine the induction of HO-1 protein expression, HepG2 cells were treated with 40 μM eckol for 24 h. As shown Figure 3A, eckol treatment induced the production of HO-1 protein in a time-dependent manner. Additionally, eckol treatment for 16 h enhanced HO-1 protein expression in a dose-dependent manner (Figure 3B). The increase HO-1 expression correlated with HO-1 mRNA levels in a dose-dependent manner in eckol treated HepG2 cells (Figure 3B). These results suggest that the eckol upregulates the expression of HO-1 at transcriptional level.

HO-1 is a key enzyme of the antioxidant defense system. Increased HO-1 activity leads to enhanced protection against free radicals produced by intrinsic or extrinsic stimuli. HO-1 is the inducible form of HO that catalyzes the conversion of heme into biliverdin, carbon monoxide, and free iron as rate-limiting enzyme [23]. Many studies have demonstrated the potent antioxidant and cytoprotective activities of heme derived metabolites produced by HO-1 [24]. Therefore, enhanced activities of HO-1 may protect HepG2 cells against possible oxidative damage. Eckol increased the expression of HO-1 in both of transcriptional and translational level of HepG2 cells (Figure 3), indicating that the compound initiate cellular events related on antioxidative and cytoprotective pathways.

### 2.3. Effect of Eckol on Nrf2 Nuclear Translocation and Activation

**Figure 4:**
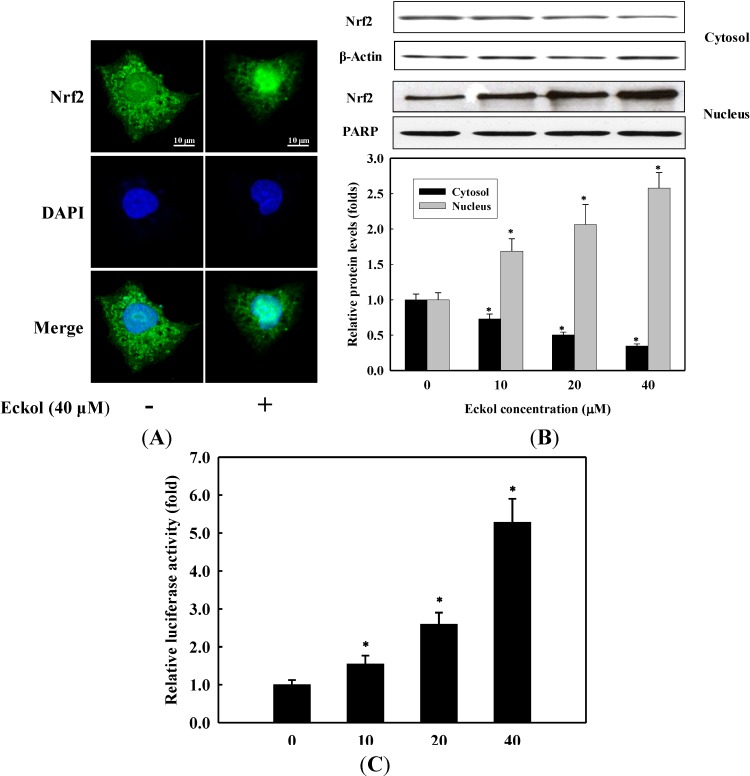
Effect of eckol on the translocation and activation of Nrf2. (**A**) Cells were pretreated with or without 40 μM eckol for 1 h. The cells were fixed and immunostained with anti-Nrf2 antibody for 2 h. The nuclei were stained with DAPI and the images were captured by confocal microscopy (×100); (**B**) Cells were treated with various concentrations of eckol for 1 h. Cytosolic and nucleic fractions were prepared and analyzed by Western blot using corresponding antibodies; (**C**) Cells transfected with ARE promoter-containing luciferase DNA were treated with various concentrations of eckol for 1 h. Values are the mean ± SD of three independent experiments. * *p* < 0.05 indicates significant differences compared to the control group.

Since HO-1 protein is primarily regulated by the transcriptional activation of Nrf2, we determined the translocation of Nrf2 from cytosol into nucleus using confocal microscope. Observation revealed that Nrf2 proteins (green) were mostly distributed in the cytoplasm of unstimulated cells. After stimulation with 40 μM eckol for 1 h, most cytoplasmic Nrf2 was translocated into the nucleus, as shown by intense Nrf2 staining in the nucleus in immunofluorescence assay (Figure 4A). To further investigate the translocation of Nrf2 into nucleus upon eckol treatment, cell lysate was separated into cytosolic and nucleic fractions, and the levels of Nrf2 in each fraction were measured by Western blot. As shown in Figure 4B, the levels of Nrf2 in cytosolic fraction were progressively reduced in a dose-dependent manner, whereas increased Nrf2 levels in nucleic fractions were observed. Considering the translocation of Nrf2 into nucleus by eckol, we next determined the effect of eckol on the promoter activity of Nrf2 in ARE/luciferase gene-transfected HepG2 cells. As presented in Figure 4C, eckol treatment significantly increased the transcriptional activity of Nrf2 (*p* < 0.05). The result indicates that eckol activates the transcriptional activity of Nrf2 via translocation into nucleus in HepG2 cells, suggesting the compound induces the expression of HO-1 through the activation of Nrf2-ARE signaling pathway.

Among the various cytoprotective enzymes, HO-1 has been considered to be an adaptive and beneficial response to oxidative stress in a wide variety of cells [25,26]. Elevated HO-1 activity is associated with an increase in the catabolism of the pro-oxidant free heme to bile pigments which are potent endogenous antioxidants [9]. There are many reports that HO-1 is induced by various phytochemicals [7,13,14], the present result is the first demonstration that eckol is a potent inducer of HO-1 expression in HepG2 cells. The regulation of HO-1 is mediated by Nrf2, which plays an essential role in the ARE-mediated expression of HO-1 and in the activation of other stress-inducible genes in response to oxidative stress [27]. Upon stimulation, Nrf2 dissociates from its cytoplasmic inhibitory protein Keap1, translocates into the nucleus and binds to ARE site [28]. Once migrated to the nucleus, Nrf2 forms heterodimers with small Maf proteins and subsequently binds to the cis-acting ARE site. This leads to the transcriptional activation of antioxidant enzymes and other cytoprotective proteins [29]. Although several studies have performed on the antioxidant effects of eckol using *in vitro* and *in vivo* models [7,15], there was no report on the ARE-mediated expression of HO-1 by eckol in HepG2 cells. In this respect, we found that eckol clearly induces the nuclear translocation and activation of Nrf2 (Figure 4), an essential process to execute its transcriptional regulation. Moreover, this observation was consistent with the increased HO-1 expression (Figure 3), suggesting that the nuclear translocation of Nrf2 by eckol might contribute to the induction of ARE-mediated HO-1 expression. This report is, to our best knowledge, the novel findings to address the functions of eckol on the expression of HO-1 through Nrf2 pathway in HepG2 cells.

### 2.4. Effect of Eckol on Phosphorylation of MAPKs and PI3K/Akt

**Figure 5:**
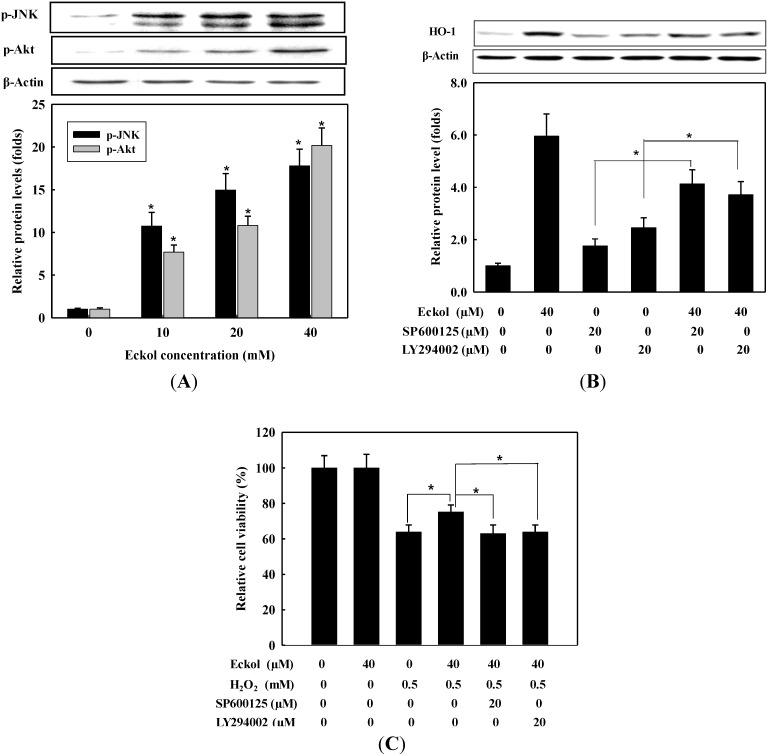
Effect of eckol on the phosphorylation of JNK and Akt in HepG2 cells. (**A**) Cells were treated with indicated concentrations of eckol for 1 h. Whole cell protein was analyzed by Western blot using anti-p-JNK and anti-pAkt; (**B**) Cells pretreated with or without SP600125 or LY294002 for 1 h were stimulated with eckol for 16 h. Whole cell protein was used for the detection of HO-1 using Western blot; (**C**) Cells pretreated with indicated concentrations of eckol or inhibitors for 1 h and then treated with 0.5 mM H_2_O_2_ for 24 h. The cell viability was determined by MTS assay kit. Values are the mean ± SD of three independent experiments. * *p* < 0.05 indicates significantly differences between groups.

To further determine the upstream signaling pathway involved in eckol-mediated Nrf2 activation and induction of HO-1, we assessed the phosphorylation of MAPKs and Akt, which are signaling proteins involved in cellular protection against oxidative stress. Phosphorylation of the proteins by eckol was assessed using Western blot. For this, cells were treated with eckol (0–40 μM) for 1 h, and cell extracts were prepared and analyzed with corresponding antibodies. As shown in Figure 5A, the levels of phosphorylated JNK and Akt were dose-dependently increased by eckol treatment, indicating the additional characteristics of eckol to activate Nrf2 via the phosphorylation of JNK and Akt. However, the phosphorylation of ERK and p38 MAPK was not changed by eckol treatment (data not shown). To further confirm the association of JNK and Akt with the expression of HO-1 through the activation of Nrf2 pathway, cells were preincubated for 1 h with SP600125 (a JNK inhibitor) and LY294002 (a PI3K/Akt inhibitor), and then stimulated with 40 μM eckol. After additional 16 h incubation, the expression level of HO-1 was determined by Western blot. As shown in Figure 5B, HO-1 expression was remarkably suppressed by the pre-treatment of a JNK or PI3K/Akt inhibitor, however, treatment with 40 μM eckol significantly induced HO-1 expression in inhibitor-treated cells (*p* < 0.05). These data confirmed additional characteristics of eckol to regulate activation s of JNK and PI3K/Akt on the expression of HO-1 via Nrf2 activation. Furthermore, we investigate whether eckol could alleviate oxidative cell death through the activation of JNK and PI3K/Akt. After incubation with eckol, JNK, or PI3K/Akt inhibitor, cells were treated with 0.5 mM H_2_O_2_ for 24 h and cell viability was determined by MTS assay. As shown in Figure 5C, SP600125 and LY294002 attenuated the protective effect of eckol against H_2_O_2_-induced cytotoxicity, suggesting the involvement of JNK and PI3K/Akt signaling in eckol-mediated HO-1 induction as well as in cytoprotection against oxidative cell death.

Increasing evidence indicates that the activation of MAPKs and Akt signaling in HepG2 cells induces ARE-mediated gene expression via an Nrf2-dependent mechanism, however, the role of MAPKs and Akt on the Nrf2 activation have shown controversial results in hepatocytes. Nrf2-mediated HO-1 expression was positively regulated by ERK and JNK signaling pathways in butylated hydroxyanisole-treated [30] and by ERK and p38 MAPK signaling pathways in pyrrolidine dithiocarbamate-treated HepG2 cells [31,32], however, p38 MAPK works as a negative regulator in Nrf2-mediated HO-1 expression [33]. Moreover, the expression of HO-1 by apo-8'-lycopenal was demonstrated with the activation of Nrf2-ARE pathways via the phosphorylation of ERK and p38 MAPK, not Akt, in HepG2 cells [34]. Of interest, cytoprotective action of eckol against oxidative stress-induced V79-4 cells was caused by Nrf2-mediated HO-1 expression via enhanced activation of ERK and Akt, not JNK and p38 MAPK, in V79- cells. Thus, a possible explanation for such contradictory observations on the regulation of Nrf2 signaling pathway may depend on the kinds of inducers and cell types as well as the relative response of signaling pathways to a given stimulus. In this study, we found that eckol activated Nrf2-ARE pathways via the phosphorylation of JNK and PI3K/Akt, not ERK and p38 MAPK. Various evidences suggest that phytochemicals are able to regulate MAPKs and Akt signaling pathways by removal of ROS with their intrinsic antioxidant activities [35], suggesting potential candidates as antioxidant or anti-inflammatory agents. The increase in Nrf2 level induced by eckol was dependent on the activation of JNK and PI3K/Akt signaling pathways because JNK and PI3K/Akt inhibitor decreased the eckol-induced accumulation of HO-1 protein. Eckol-treated cells clearly induced the translocation of Nrf2 into nucleus, and the activated Nrf2 might play a role in enhanced HO-1 production, leading increased cell viability in H_2_O_2_-treated HepG2 cells demonstrated by Figure 5C.

## 3. Experimental Section

### 3.1. Chemicals

MEM (minimum essential medium), penicillin-streptomycin mixture, 0.25% trypsin-EDTA, non-essential amino acid, sodium pyruvate, fetal bovine serum (FBS) were purchased from Gibco-BRL Life Technologies (Grand Island, NY, USA). CellTiter 96 AQueous One Solution Cell Proliferation assay kit was purchased from Promega (Madison, WI, USA). Cignal antioxidant response reporter assay kit was obtained from Qiagen (Valencia, CA, USA). Lipofectamine/Plus, Alexa Fluor^®^ 488-conjugated secondary antibody, and TRIzol were obtained from Invitrogen (Carlsbad, CA, USA). The enhanced chemiluminescence (ECL) detection kit was purchased from GE Healthcare Bio-Science (Piscataway, NJ, USA). The antibodies against ERK, JNK, p38 MAPK, Akt, HO-1, poly(ADP-ribose) polymerase (PARP), Nrf2, β-actin and horseradish peroxidase-conjugated secondary antibodies were purchased from Santa Cruz Biotechnology (Santa Cruz, CA, USA). 2',7'-Dichlorofluorescein diacetate (DCF-DA), phenylmethylsulfonyl fluoride (PMSF), bovine serum albumin (BSA), N-acetylcysteine (NAC), 4',6-diamidino-2-phenylindole (DAPI), and dimethyl sulfoxide (DMSO) were purchased from Sigma Chemical Co. (St. Louis, MO, USA). GSH assay kit was obtained from Cayman Chemical Co. (Ann Arbor, MI, USA).

### 3.2. Isolation of Eckol

The isolation eckol was performed according to the method of Lee *et al.* (22). Ethanolic extract of *E. stolonifera* was partitioned with *n*-hexane, ethyl acetate (EtOAc), *n*-butanol solvents in sequence. An aliquot of EtOAc fraction was subjected to preparative size exclusion column of Asahipak GS-310 (500 × 20 mm, Showa Denko, Tokyo, Japan). An exclusion HPLC apparatus consisted of a pump (Shimadzu LC-6AD, Shimadzu Co., Tokyo, Japan), a photodiode array detector (Shimadzu SPD-M20A), an online degasser (Shimadzu DUG-20A3), an autosampler (SIL-20A), a fraction collector (Shimadzu FRC-10A), a system controller (CBM-20A), and a Shimadzu LCsolution (ver. 1.22sp). EtOAc fraction was chromatographed on an Asahipak GS-310 column eluting with methanol at a flow rate of 5.0 mL/min and monitored at 245 nm. The fraction was separated into five subfractions (GS1−GS5). The GS4 fraction showing high antioxidant activity based on DPPH radical scavenging activity was chromatographed over a Shim-pack PREP-ODS column (5 μm, 100 Å, 250 mm × 20 mm, Shimadzu Co.). A preparative ODS HPLC system was similar to the exclusion HPLC system except for a binary pump (Shimadzu LC-6AD) and a column oven (35 °C, Shimadzu CTO-20A). The separation of the GS4 fraction was conducted using a mobile phase of 0.1% formic acid in water (solvent A) and 0.1% formic acid in acetonitrile (solvent B). The elution profile consisted of a linear gradient from 20% to 100% of solvent B for 60 min and re-equilibration of the column with 20% solvent B for 10 min. The flow rate was 7.0 mL/min, and detection was performed at 245 nm. The fraction gave six subfractions (GS4-ODS1−GS4-ODS6). GS4-ODS4 fraction was further purified by the same HPLC system with Luna RP-18 [Luna C18(2), 5 μm, 250 × 10 mm, Phenomenex, Torrance, CA, USA] and with the same mobile phase systems at a flow rate of 3.0 mL/min. Eckol (Figure 1) was identified by its ^1^H and ^13^C-NMR spectra based on the previous data [21]. Purity was determined by LC/MS analysis (>99.0%).

### 3.3. Cell Culture and Cell Viability Assay

HepG2 cells (ATCC, Rockville, MD, USA) were cultured at 37 °C in MEM supplemented with 10% FBS, non-essential amino acid, sodium pyruvate, and penicillin-streptomycin (100 U/mL penicillin and 100 μg/mL streptomycin in 0.85% saline) in a humidified atmosphere of 5% CO_2_. Cell viability was determined by 3-(4,5-dimethyl-2-yl)-5-(3-carboxymethoxyphenyl)-2-(4-sulfophenyl)-2*H*-tetrazolium (MTS) assay using CellTiter96 AQueous One Solution Cell Proliferation assay kit according to the manufacturer’s instructions. HepG2 cells were inoculated at density of 2.0 × 10^4^ cells/well into 96-well plate and treated with 40 μM eckol for 24 h. The cultured medium was removed and replaced by 95 μL of fresh culture medium and 5 μL of MTS solution. After 1 h, the absorbance was determined using microplate reader (GloMax^®^-Multi Detection System, Promega) at 490 nm.

### 3.4. Measurement of Intracellular ROS

The intracellular ROS scavenging activity of the eckol was measured using the oxidant-sensitive fluorescent probe DCF-DA. HepG2 cells (2 × 10^4^ cells/well) seeded in 96-well plates were cultured for 16 h. The cells were pretreated with various concentration of eckol. After 2 h, cells stimulated with 0.5 mM H_2_O_2_ at a final concentration were incubated for an additional 30 min. After washing with phosphate-buffered saline (PBS) twice, the cells were treated with 20 μM DCF-DA for 30 min at 37 °C. The fluorescence intensity was measured at excitation wavelength of 485 nm and emission wavelength of 528 nm using fluorescence microplate reader (Dual Scanning SPECTRAmax, Molecular Devices Co., Sunnyvale, CA, USA).

### 3.5. Measurement of Reduced Glutathione

HepG2 cells cultured in 12-well plates (1 × 10^6^ cells/well) were treated with indicated concentration of eckol for 2 h, since the change of antioxidant enzyme activities were detectable after 2 h eckol treatment. The cells were washed twice with PBS and lysed in complete radioimmunoprecipitation assay buffer containing 50 mM Tris-HCl (pH 7.4), 150 mM NaCl, 1% Nonidet P-40, 0.5% sodium deoxycholate, 0.1% SDS, 2 mM PMSF, 1 mM sodium orthovanadate, and 2 μg/mL of each aprotinin, leupeptin and pepstatin on ice for 30 min and then centrifuged at 10,000× *g* for 15 min. The supernatant was transferred and stored at −70 °C until required. GSH levels were measured using the GSH assay kit according to the manufacturer’s instruction.

### 3.6. Preparation of Cell Lysates and Western Blotting

HepG2 cells were washed twice with PBS and homogenized in RIPA buffer on ice for 30 min. After centrifugation at 18,000× *g* for 10 min, aliquots of protein (30 μg) in supernatant were resolved by sodium dodecyl sulfate-polyacrylamide gel electrophoresis (SDS-PAGE) and transferred onto nitrocellulose membranes (Millipore, Bedford, MA, USA). The membranes were washed with Tris-buffered saline (10 mM Tris-HCl, 150 mM NaCl, pH 7.5) supplemented with 0.05% (v/v) Tween 20 (TBST) followed by blocking with TBST containing 5% (w/v) non-fat dried milk. The membranes were incubated overnight with antibodies against HO-1, pJNK, Nrf2, PARP, or β-actin at 4 °C. After washing with TBST, the membranes were then exposed to secondary antibodies coupled to horseradish peroxidase for 2 h at room temperature. The membranes were washed three times with TBST at room temperature. Immunocomplexes were detected by ECL reagents. Equal protein loading was assessed by the expression level of β-actin or PARP. Densitometric analysis of the data obtained from at least three independent experiments was performed using cooled CCD camera system EZ-Capture II and CS analyzer ver. 3.00 software (ATTO Co., Tokyo, Japan).

### 3.7. Transfection and Luciferase Assay

One hundred nanograms of positive control mixture including ARE promoter/firefly luciferase reporter construct and Renilla luciferase construct (40:1) was transiently transfected into 1 × 10^6^ HepG2 cells using Lipofectamine^TM^ Plus reagents for 24 h. Cells were then treated with 0 to 40 μM eckol for 6 h. Each well was then washed twice with cold PBS and suspended in 100 μL of lysis buffer (0.5 mM HEPES, pH 7.8, 1% Triton N-101, 1 mM CaCl_2_, and 1 mM MgCl_2_) and used for the measurement of luciferase activity using a luciferase assay kit. Luminescence was measured on a top counter microplate scintillation and luminescence counter in single photon counting mode for 0.1 min/well, following a 5 min adaptation in the dark. The luciferase activity was normalized with expression of negative control mixture including non-inducible firefly luciferase reporter construct and luciferase construct (40:1).

### 3.8. Isolation of Nuclear and Cytosolic Fraction

Conditioned cells were washed twice with ice-cold PBS, scraped into 1 mL of cold PBS, and pelleted by centrifugation at 300× *g* for 5 min. Cell pellets were resuspended in 300 μL of hypotonic buffer (10 mM HEPES/KOH, 10 mM KCl, 2 mM MgCl_2_, 0.1 mM EDTA, 1 mM DTT, and 0.5 mM PMSF, pH 7.9), and stood on ice for 15 min. After mixing for 10 sec, homogenates were centrifuged at 13,000× *g* for 1 min. The supernatant was collected as cytosolic fraction. The pellet was gently resuspended in 30 μL of complete lysis buffer (50 mM HEPES/KOH, 50 mM KCl, 1 mM DTT, 300 mM NaCl, 0.1 mM EDTA, 10% glycerol, and 0.5 mM PMSF, pH 7.9), and centrifuged at 13,000× *g* for 20 min at 4 °C. The supernatant was used as nuclear fraction.

### 3.9. Immunofluoresence Analysis

To analyze nuclear localization of Nrf2 in HepG2 cells, cells were maintained on glass coverslips for 24 h. After stimulation with 40 μM eckol, cells were fixed in 4.0% (w/v) paraformaldehyde in PBS for 15 min at room temperature, and permeabilized with 0.5% (v/v) Triton X-100 in PBS for 10 min. Permeabilized cells were washed with PBS, and blocked with 3% (w/v) BSA in PBS for 30 min. Thereafter, cells were incubated in an anti-Nrf2 monoclonal antibody diluted in 3% BSA in PBS for 1 h, rinsed three times with PBS, and incubated in Alexa Fluor 488-conjugated secondary antibody diluted in 3% BSA in PBS for 1 h. Cells treated with 2 μg/mL DAPI were viewed with LSM700 Laser scanning confocal microscope (Carl Zeiss, Oberkochen, Germany).

### 3.10. Statistical Analysis

All data are expressed as the mean ± SD Data were analyzed using one-way ANOVA. Differences were considered significant of *p* < 0.05. All analyses were performed using SPSS for Windows, version 10.07 (SPSS, Chicago, IL, USA).

## 4. Conclusions

In conclusion, we have demonstrated that eckol induced the expression of HO-1 in HepG2 cells. The induction of HO-1 is associated with the ability of eckol to activate transcriptional factor Nrf2 through the activation of JNK and PI3K/Akt signaling pathways. The findings provide novel mechanisms underlying eckol in cytoprotection and chemoprevention in HepG2 cells.
